# Structural insights into immune escape at killer T cell epitope by SARS-CoV-2 Spike Y453F variants

**DOI:** 10.1016/j.jbc.2024.107563

**Published:** 2024-07-11

**Authors:** Shasha Deng, Zhihao Xu, Meihua Wang, Jing Hu, Zhuan Liu, Fang Zhu, Peiyi Zheng, Arnaud John Kombe Kombe, Hongliang Zhang, Songquan Wu, Tengchuan Jin

**Affiliations:** 1 Department of Obstetrics and Gynecology, The First Affiliated Hospital of USTC, Division of Life Sciences and Medicine, Center for Advanced Interdisciplinary Science and Biomedicine of IHM, University of Science and Technology of China, Hefei, Anhui, P.R. China; 2 Laboratory of Structural Immunology, Key Laboratory of Immune Response and Immunotherapy, Division of Life Sciences and Medicine, University of Science and Technology of China, Hefei, China; 3 College of Medicine, Lishui University, Lishui, China; 4 Institute of Health and Medicine, Hefei Comprehensive National Science Center, Hefei, Anhui, China; 5 Biomedical Sciences and Health Laboratory of Anhui Province, University of Science & Technology of China, Hefei, China; 6 Clinical Research Hospital of Chinese Academy of Sciences (Hefei), University of Science and Technology of China, Hefei, China

**Keywords:** SARS-CoV-2, immune escape, mutations, CD8^+^ T cell epitope, HLA-A24, TCR-pHLA

## Abstract

CD8^+^ T cell immunity, mediated by human leukocyte antigen (HLA) and T cell receptor (TCR), plays a critical role in conferring immune memory and protection against viral pathogens. The emergence of SARS-CoV-2 variants poses a serious challenge to the efficacy of current vaccines. Whereas numerous SARS-CoV-2 mutations associated with immune escape from CD8^+^ T cells have been documented, the molecular effects of most mutations on epitope-specific TCR recognition remain largely unexplored. Here, we studied an HLA-A24-restricted NYN epitope (Spike_448-456_) that elicits broad CD8^+^ T cell responses in COVID-19 patients characterized by a common TCR repertoire. Four natural mutations, N450K, L452Q, L452R, and Y453F, arose within the NYN epitope and have been transmitted in certain viral lineages. Our findings indicate that these mutations have minimal impact on the epitope's presentation by cell surface HLA, yet they diminish the affinities of their respective peptide-HLA complexes (pHLAs) for NYN peptide-specific TCRs, particularly L452R and Y453F. Furthermore, we determined the crystal structure of HLA-A24 loaded with the Y453F peptide (NYNYL**F**RLF), and subsequently a ternary structure of the public TCR^NYN-I^ complexed to the original NYN-HLA-A24 (NYNYLYRLF). Our structural analysis unveiled that despite competent presentation by HLA, the mutant Y453F peptide failed to establish a stable TCR-pHLA ternary complex due to reduced peptide: TCR contacts. This study supports the idea that cellular immunity restriction is an important driving force behind viral evolution.

SARS-CoV-2 infection elicits broad activation of cytotoxic CD8^+^ T lymphocytes (CTLs), which is one of the major correlates of antiviral protection. Circulating T cells are strongly related to the pathogenesis of COVID-19 (1), and there is evidence that mild convalescents exhibit a higher proportion of multifunctional CTLs compared to those with severe symptoms (2). SARS-CoV-2-specific CTLs appear in the blood of patients with COVID-19 before recovery, displaying a broad range of cytotoxic molecules such as granzyme B, perforin, and IFN-γ (3, 4, 5). Following the initial dose of the mRNA BNT162b2 vaccine, CTL responses are activated within 1 week, while neutralizing antibodies were not fully induced at this time, indicating that early vaccine-induced protection may primarily depend on T cells (6). Both *in vivo* and *in vitro* studies have consistently demonstrated significant CTL activations during SARS-CoV-2 infection (5, 7, 8, 9).

The emergence of SARS-CoV-2 variants has raised concerns about cellular immune escape. Mutations at certain positions may impair epitopes loading onto major histocompatibility complex (MHC, HLA in humans) molecules or disrupt specific recognition by T cell receptors (TCRs) (10, 11). Our previous study has shown that SARS-CoV-2 variants harbor multiple mutations within CTL epitopes, such as Y144del, L452R, and K417N/T on the Spike protein, significantly reducing CD8^+^ T cell activations in COVID-19 convalescents and vaccinated individuals (12). In one report, the P272L mutation at the center of the dominant YLQ epitope (Spike_269-277_: YLQPRTFLL), present in multiple lineages, resulted in the incapable recognition by over 175 TCRs in cohorts of convalescents and vaccinees (13). In another report, the T1006I mutation within the RLQ epitope (Spike_1000-1008_, RLQSLQTYV) has been implicated in disrupting specific TCR binding and subsequent T cell responses (14). The crystal structures of four of YLQ-HLA-A2 and three of RLQ-HLA-A2 bound their respective TCRs unveiled the molecular basis underlying recognition loss or reduction due to these mutations (11, 14, 15, 16, 17). However, structural information regarding the ternary complex of SARS-CoV-2 peptide-HLA (pHLA) complexes bound to peptide-dependent TCRs remains highly limited. The currently available structures predominantly concentrate on the HLA-A∗02:01 (A02) restricted YLQ and RLQ epitopes (14, 15, 16, 17). The extent to which SARS-CoV-2 mutations may affect the epitope presentation through MHC-I remains to be determined.

The size, frequency, and public nature of TCR repertoire responding to antigenic epitopes can provide an in-depth understanding of successful and failed immune responses. During the pandemic, public and diverse human TCRs targeting the Spike epitopes have been observed in COVID-19 convalescents (18). Public TCR repertoires often exhibit biases, showing a preference for specific TCRα chain Variable (TRAV) or TCRβ chain Variable (TRBV) segments (19). For example, a large majority (>85%) of YLQ-specific TCRs utilize nearly identical TRAV12-1 or TRAV12-2 segments with the public CDR3 motifs that are shared across individuals (11, 18, 20). These public TCRs are thought to have a selective advantage, primarily composed of germline-encoded sequences (21). Opposite to the YLQ epitope, the RLQ epitope is recognized by private TCRs with a variety of unrelated α/β chain pairs (18). Such TCR privacy relies on highly variable CDR3α and CDR3β loops to recognize pHLA complexes, reducing the likelihood of identical VJ (α chain) or VDJ (β chain) recombination in different individuals (14).

HLA-A∗24:02 (A24) is a highly prevalent genotype, second only to HLA-A02 (22, 23, 24). The HLA-A24-restricted NYN epitope (Spike_448-456_, NYNYLYRLF) elicits strong and broad CTL responses in antiviral immunity (7, 12, 25, 26, 27). Notably, mutations such as L452R and Y453F within this epitope have been reported to facilitate immune escape from HLA-A24-restricted cellular immunity (28). In this study, we found that the L452R and Y453F mutations significantly reduce the interactions between their pHLAs and epitope-specific TCRs. Concurrently, we determined the crystal structure of the Y453F peptide bound to HLA-A24, as well as a ternary structure of the epitope-specific TCR^NYN-I^ with the original NYN-HLA-A24 complex, highlighting that some dominant mutations like Y453F may evade CTL responses by disrupting the formation of stable TCR-pHLA ternary complexes.

## Results

### Emergence of mutations in the HLA-A24-restricted NYNYLYRLF CD8^+^ T cell epitope

**Figure 1:**
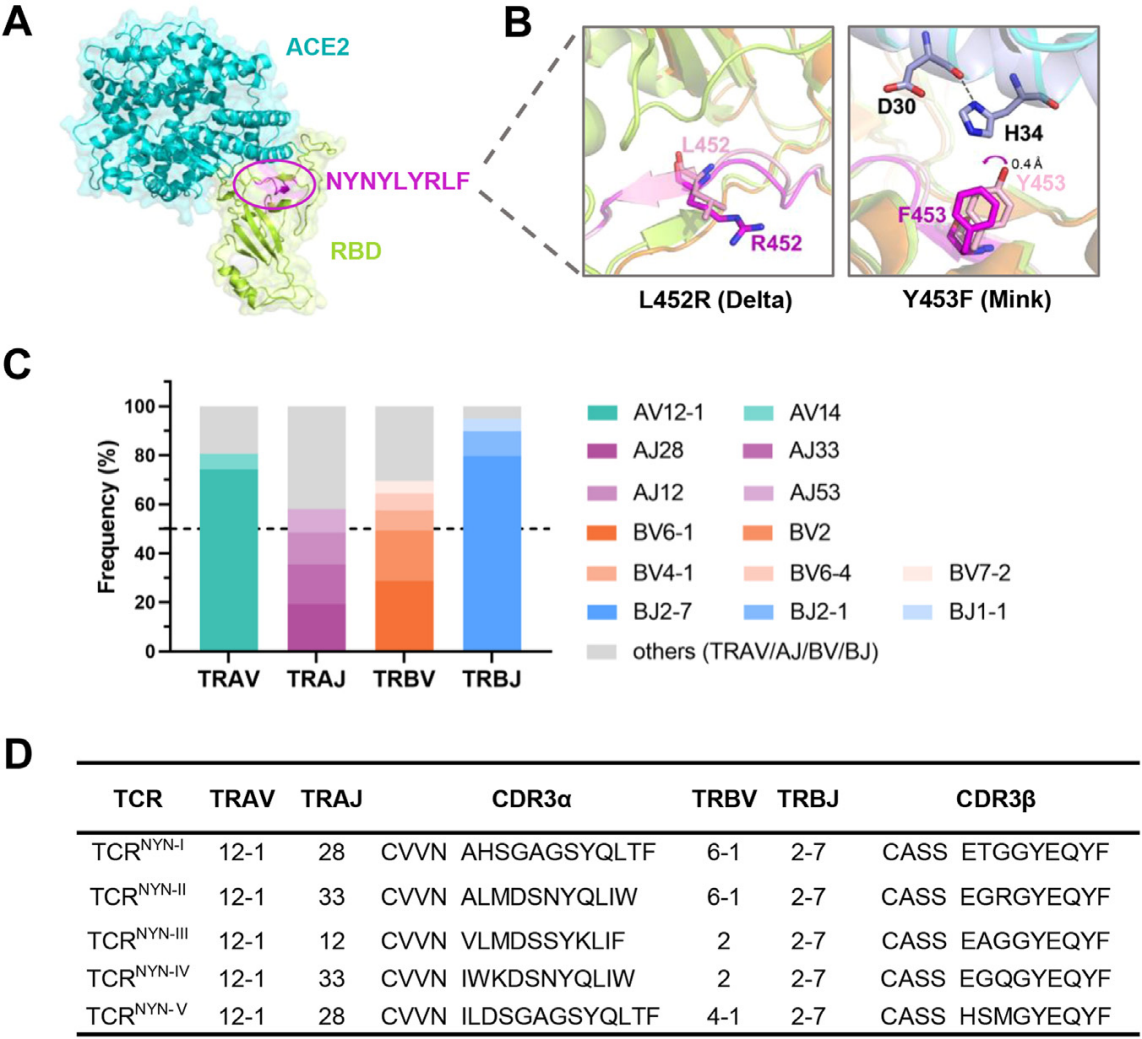
**Nonsynonymous mutations are detected in the SARS-CoV-2 NYN epitope.***A*, localization of the NYN-peptide on the original SARS-CoV-2 Spike protein (ID: 7KMS). Spike-RBD, ACE2, and Y453F peptide are shown in *limon*, *cyan* and *magenta*, respectively. *B*, detailed conformations of the L452R peptide and Y453F peptide superimposed on the Spike structure of the original SARS-CoV-2 (ID: 6LZG), along with the L452R-Delta (ID: 7W92) or Y453F-Mink (ID: 7EKH) variants. The RBD is shown in *limon* (WT-RBD) or *orange* (L452R-Delta-RBD and Y453F-Mink-RBD). ACE2 is depicted in *cyan* (WT-ACE2) or *light blue* (Y453F-Mink-ACE2). Carbon atoms of the peptides are in *light pink* (WT peptide) or *magenta* (L452R peptide or Y453F-peptide); nitrogen atoms are *blue*; oxygen atoms are *red*. *C*, composition of NYN-specific TCRs. Only TCRα (TRAV, TRAJ) and TCRβ (TRBV, TRBJ) genes with frequencies > 5% are shown. *D*, five high-frequency paired NYN-specific TCRs (TCR^NYN-I^, TCR^NYN-II^, TCR^NYN-III^, TCR^NYN-IV^, TCR^NYN-Ⅴ^) with different CDR3 motifs are selected for protein expression.

The Spike receptor-binding domain (RBD) of the SARS-CoV-2 genome is essential for viral entry, mediated by its binding to angiotensin-converting enzyme 2 (ACE2) (29, 30). Among the Spike epitopes, the NYN epitope is particularly significant due to its structural location at the ACE2-RBD interface (Fig. 1*A*). Importantly, we identified a mutation hotspot on the NYN epitope in the SARS-CoV-2 sequences collected from the public database GISAID (31), including mutations N450K, L452Q, L452R, and Y453F (Fig. S1*A*). The L452R mutation first emerged in the Delta variant, which was the dominant variant before the Omicron pandemic. Previous studies have pointed out that the hydrophobic leucine mutates into a basic arginine with an elongated side chain (28, 32), which may affect the binding of antibodies (Fig. 1*B*). Over the past 2 years, L452R has consistently ranked among the top 20 mutations in the Omicron-RBD protein (Fig. S1*B*). The Y453F mutation is another dominant mutation, initially detected in the human-to-mink transmitted variants (Mink). The structure of Mink-Y453F RBD-hACE2 indicates that the mutation causes a 0.4 Å shift of the phenyl group of F453 (Fig. 1*B*), enhancing the Spike protein's affinity for ACE2 and facilitating host adaptation (33, 34). In contrast, mutations L452Q and N450K are less frequent and have attracted fewer concerns. L452Q has been reported to increase the effective reproduction number of Omicron BA2 (35), and N450K may diminish the neutralization capacity of monoclonal antibodies and convalescent serum (36). Notably, binding prediction of the wild type (WT) and mutant peptides to HLA-A24 revealed strong peptide binding to the MHC-I molecule, with comparable binding levels of NetMHCpan4.1 (37) % ranks (<0.5) (Fig. S1*C*).

At the molecular level, viral mutations may compromise T cell activation in two primary ways: by directly reducing peptide-HLA complex formation during the antigen presentation stage and by interfering with specific TCR recognition. To corroborate our hypothesis, we analyzed the sequence composition of NYN peptide-specific TCRs (NYN-TCRs) based on the data from the public databases VDJdb (38) and IEDB (39). We found that the predominant Vα/Jβ combinations were TRAV12-1 and TRBJ2-7, occurring at frequencies of 74% and 80%, respectively (Fig. 1*C*). A similar bias for TRAV12-1 was also observed in the public YLQ-specific TCR repertoire (14). For our subsequent experiments, we selected five high-frequency TCRαβ combinations with diverse CDR3 sequences (Fig. 1*D*). The pairing of these TCRα and TCRβ chains was based on their co-occurrence in samples from the same patients and their relative frequencies (38, 40).

### Interaction of NYN-specific TCRs with original epitope and epitope variants

**Figure 2:**
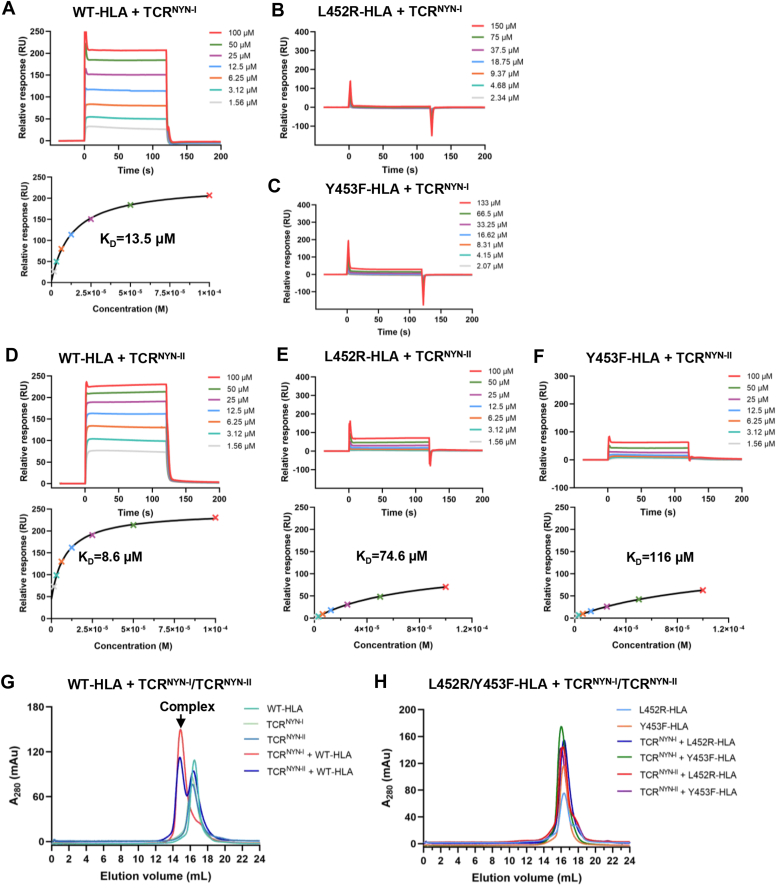
**L452R and Y453F variants lead to weakened TCR: pHLA interactions.***A*, (*upper*) WT-HLA at concentrations of 1.56, 3.12, 6.25, 12.5, 25, 50 and 100 μM was injected over immobilized TCR^NYN-I^. (*lower*) Fitting curves for equilibrium binding that resulted in a K_D_ of 13.5 μM. *B*, L452R-HLA at concentrations of 2.34, 4.68, 9.37, 18.75, 37.5, 75, and 150 μM was injected over immobilized TCR^NYN-I^. *C*, Y453F-HLA at concentrations of 2.07, 4.15, 8.31, 16.62, 33.25, 66.5 and 133 μM was injected over immobilized TCR^NYN-I^. *D*, (*upper*) WT-HLA at concentrations of 1.56, 3.12, 6.25, 12.5, 25, 50 and 100 μM was injected over immobilized TCR^NYN-II^. (*lower*) Fitting curves for equilibrium binding that resulted in a K_D_ of 8.6 μM. *E* and *F*, (*upper*) L452R-HLA or Y453F-HLA at concentrations of 1.56, 3.12, 6.25, 12.5, 25, 50, and 100 μM were injected over immobilized TCR^NYN-II^. (*lower*) Fitting curves for equilibrium binding that resulted in K_D_s of 74.6 μM and 116 μM, respectively. Each SPR experiment was conducted with two biological replicates, and the replicate results are shown in Fig. S3. *G*, size-exclusion chromatography (SEC) assays of WT-HLA protein with TCR^NYN-I^ or TCR^NYN-II^. *H*, SEC assays of mutant pHLAs (L452R-HLA, Y453F-HLA) with TCR^NYN-I^ or TCR^NYN-II^.

To assess the potential effects of viral mutations on the formation of TCR-pHLA ternary complexes, five NYN-TCRs (TCR^NYN-Ⅰ^, TCR^NYN-Ⅱ^, TCR^NYN-Ⅲ^, TCR^NYN-Ⅳ^, TCR^NYN-Ⅴ^) and five pHLAs (WT-HLA, N450K-HLA, L452Q-HLA, L452R-HLA, Y453F-HLA) were expressed, refolded, and purified *via* established procedures (12, 14). We initially employed enzyme-linked immunosorbent assay (ELISA) to detect the bindings between these pHLAs and TCRs. Mutant pHLAs exhibited lower affinities with all five NYN-TCRs compared to that of WT-HLA, suggesting that four mutations weakened the binding interactions (Fig. S2*A*). Next, TCR^NYN-I^ and TCR^NYN-II^ were immobilized, and equilibrium dissociation constants (K_D_s) for each pHLA were measured using surface plasmon resonance (SPR). TCR^NYN-I^ and TCR^NYN-II^ bound to WT-HLA with K_D_s of 13.5 μM and 8.6 μM, respectively (Fig. 2, *A* and *D*), which fall into high-functional characteristic TCRs avidity for microbial antigens, ranging from 1 μM to 50 μM of K_D_s (41). However, mutations L452R and Y453F resulted in significant reductions in pHLA affinities for TCRs, with no measurable binding observed between their pHLAs and TCR^NYN-I^ (Fig. 2, *B* and *C*). Meanwhile, L452R-HLA and Y453F-HLA bound to TCR^NYN-II^ with K_D_s of 74.6 μM and 116 μM, representing 8.7-fold and 13.5-fold affinity reductions compared to WT-HLA, respectively (Fig. 2, *E* and *F*). In contrast, mutations N450K and L452Q showed moderate impacts on affinities, with K_D_s of 19.7 μM (N450K-HLA:TCR^NYN-I^), 57.1 μM (L452Q-HLA:TCR^NYN-I^), 10.9 μM (N450K-HLA:TCR^NYN-II^), and 30.6 μM (L452Q-HLA:TCR^NYN-II^), corresponding to 1.5-fold, 4.2-fold, 1.3-fold, and 3.6-fold reductions relative to WT-HLA, respectively (Fig. S2, *B*–*E*). To ensure the robustness and reproducibility of our findings, each SPR experiment was conducted with two independent biological replicates (Fig. S3). The results of replicate measurements consistently showed no significant differences, collectively indicating that the mutations reduced the binding affinities between pHLAs and TCRs (Table S1).

Additionally, size exclusion chromatography (SEC) was applied to evaluate the formation of TCR-pHLA complexes in protein solution in *vitro*. After incubating TCR^NYN-I^ or TCR^NYN-II^ with pHLA proteins in pairs, TCR-pHLA complexes were eluted at ∼15 ml owning to increased molecular size (∼105 kDa). We observed that WT-HLA, N450K-HLA, and L452Q-HLA could be complexed with both TCR^NYN-I^ and TCR^NYN-II^ (Fig. S4, *A*–*E*), but no TCR-pHLA complex was detected for L452R-HLA and Y453F-HLA mutants (Fig. 2, *G* and *H*). These results suggest that, on average, the substitutions N450K and L452Q are tolerated, in agreement with the SPR results. In sharp contrast, TCR^NYN-I^ and TCR^NYN-II^ are highly sensitive to the L452R and Y453F variants. Our data revealed that drastic reduction of TCR: pHLA interaction makes unlikely efficient L452R and Y453F recognition by T cells with peptide-dependent TCRs, which was also consistent with the possible role of the L452R and Y453F variants in evading HLA-A24-restricted cellular immunity (28).

### Structural comparison of NYNYLYRLF-HLA-A24 and NYNYLFRLF-HLA-A24

**Figure 3:**
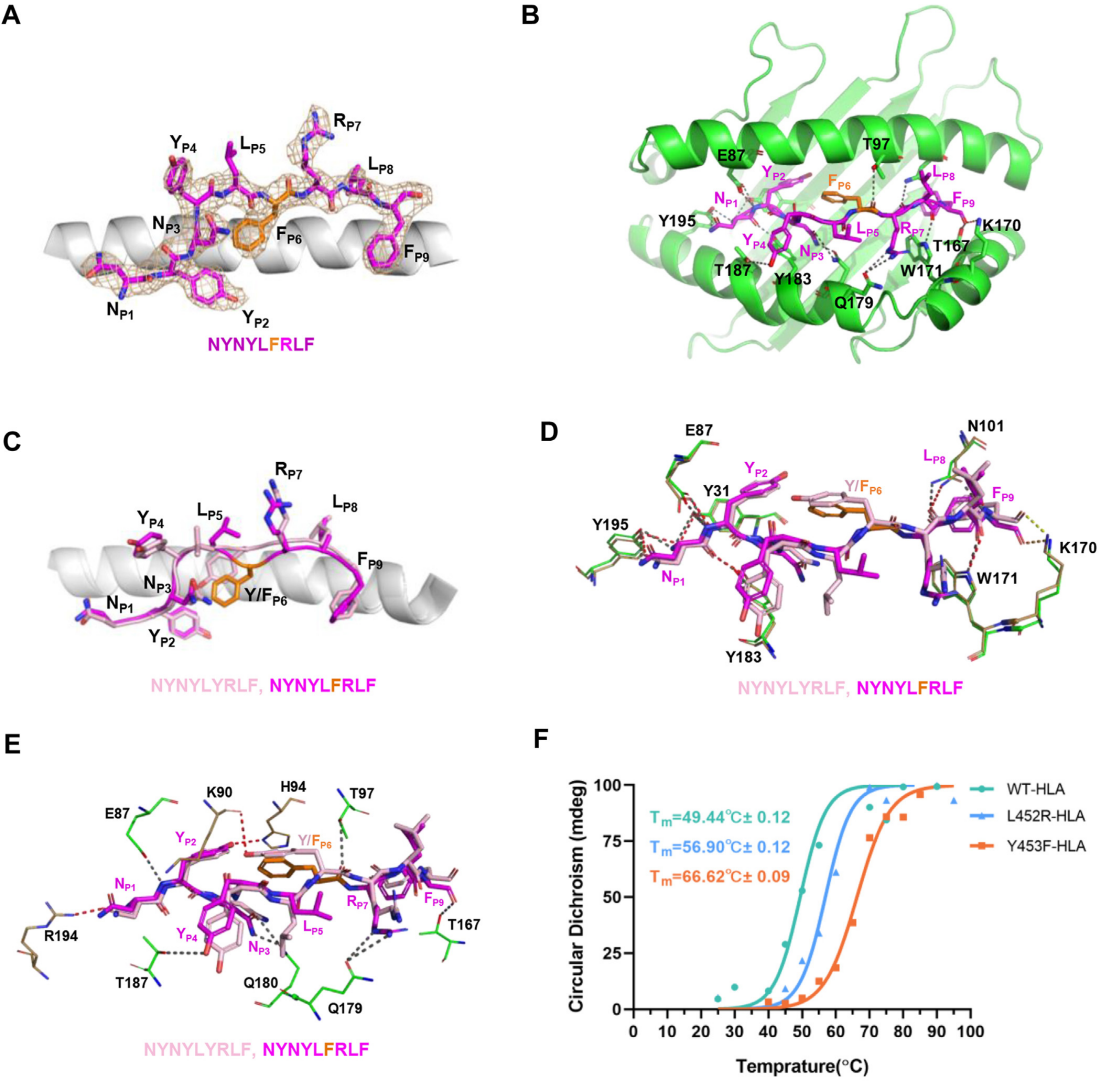
**The structures of Y453F-HLA-A24.***A*, electron density map (2Fo-Fc) of the Y453F peptide (NYNYL**F**RLF) in the crystal structure of Y453F-HLA-A24. Carbon atoms of the peptides are *magenta* and the mutation site of P6-Phe is highlighted in *orange*; nitrogen atoms are *blue*; oxygen atoms are *red*. *B*, structural overview of the interactions between HLA-A24 and the Y453F peptide. To accurately identify residues on the gene, we number them by adding 24 to the structural residue numbers of the HLA-A∗24:02-α chain. This adjustment accounts for the 24-residue intracellular segment of the HLA-A∗24:02-α chain. Carbon atoms of the Y453F peptide are *magenta*. HLA-A24 is *green*. Hydrogen bonds and salt bridges are represented by *grey* and *orange* dashes, respectively. *C*, comparison of contacts formed by WT- or Y453F-peptide with HLA-A24 cleft. Carbon atoms of the WT and Y453F peptides are light *pink* and *magenta*, respectively. HLA-A24 is *grey*. *D*, identical bonding interactions between WT- and Y453F-peptides with HLA-A24. Hydrogen bonds are represented in *red* (WT-peptide) or *grey* (Y453F-peptide), and salt bonds are shown in *yellow* (WT-peptide) or *orange* (Y453F-peptide). HLA-A24 is shown in sand (WT-HLA-A24) or *green* (Y453F-HLA-A24). *E*, distinct interactions of WT- and Y453F-peptides with HLA-A24. *F*, thermal denature fitting curves of WT-HLA, L452R-HLA, and Y453F-HLA by CD spectrum. Each CD experiment was conducted with two biological replicates, and the replicate results are shown in Fig. S4*F*. The data were fitted by Prism software.

To elucidate the basis of the mutant peptide presentation by HLA-A24, we determined the structure of Y453F-HLA-A24 (NYNYL**F**RLF) at a resolution of 2.5 Å (Table S2). Clear and continuous electron densities extend across the entire length of the MHC-bound peptide, allowing confident identification of all peptide residues (Fig 3*A*). The structure revealed the strong bindings between HLA-A24 and the Y453F peptide. The peptide backbone was bound to HLA-A24 *via* seventeen hydrogen bonds and an additional salt bridge between Lys170 and P9-Phe of the peptide (Fig. 3*B*) (Table S3). Previously, we resolved the crystal structure of NYN-HLA-A24 loaded with the original NYN peptide (12). Comparing the structures of NYNYLYRLF-HLA-A24 (ID: 7F4W (12)) and NYNYL**F**RLF-HLA-A24, we observed a dramatic change in the conformation of the peptide, with a root-mean-square difference (RMSD) of 0.80 Å (Fig. 3*C*). The most substantial deviation occurred at the central portion of the bound peptide, where the α-carbons of mutation site P6-Phe shifted by 1.7 Å (Fig. 3*C*). Both the WT and Y453F peptides were anchored to the HLA-A24 cleft through a conventional orientation involving nine hydrogen bonds and one salt bridge formed by P1-Asn, P2-Tyr, P7-Arg, P8-Leu, and P9-Phe with HLA-A24 (Fig. 3*D*). Interestingly, compared with the WT peptide, the Y453F peptide establishes more bond interactions with HLA-A24 helix *via* P2-Tyr, P3-Asn, P4-Tyr, P6-Phe, P7-Arg, and P9-Phe, forming eight hydrogen bonds with E87, T97, T167, Q179, Q180, and T187 (Fig. 3*E*). The solvent-exposed side chains of P4-Tyr, P5-Leu, and P7-Arg of the peptide were positioned away from the peptide-binding groove, composing a moderate surface for potential interactions with TCRs. We subsequently utilized cell-free circular dichroism (CD) to assess the thermal stability of pHLA complexes. Compared to WT-HLA, both mutant L452R-HLA and Y453F-HLA exhibited enhanced stability, as evidenced by increased melting temperatures (Figs. 3*F*, S4*F*). This finding contrasts with previous research suggesting reduced or abolished MHC-I binding with certain mutant peptides (13). Our results indicate that the mutations L452R and Y453F actually promote more stable binding of peptides to the HLA-A24 groove.

### Overview of TCR^NYN-I^-NYN-HLA-A24 complex and the interaction of TCR^NYN-I^ with HLA-A24

**Figure 4:**
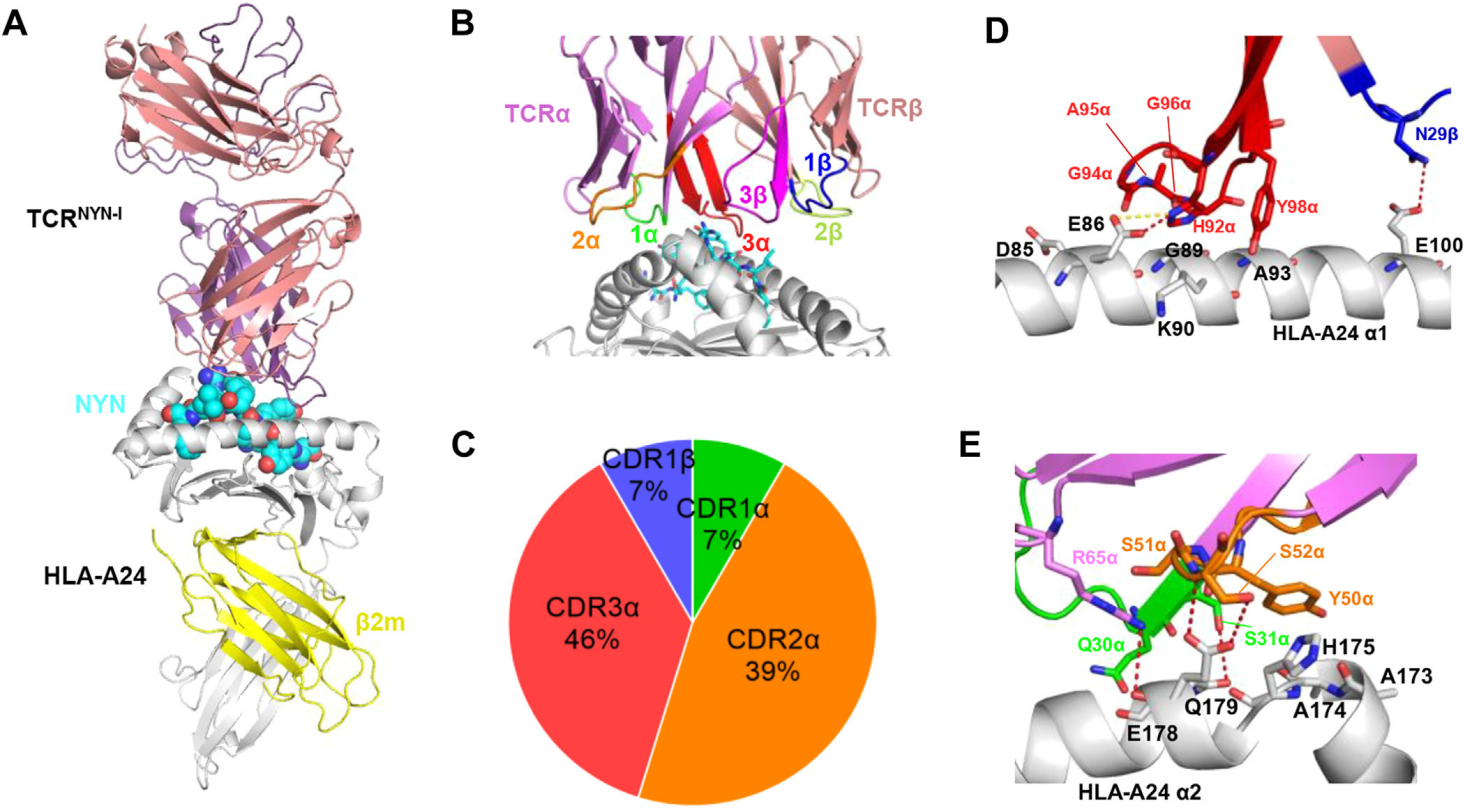
**3D structure of TCR**^**NYN-I**^**-NYN-HLA-A24 complex.***A*, side view of TCR^NYN-I^-NYN-HLA-A24 (ribbon diagram). TCRα chain, *violet*; TCRβ chain, salmon; HLA-A24 heavy chain, *gray*; β2m, *yellow*. The NYN peptide is shown as *cyan* spheres. *B*, detailed overview of the TCR^NYN-I^ is represented as cartoon on the *top* of the NYN-peptide presented by the HLA-A24. CDR1α, *green*; CDR2α, *orange*; CDR3α, *red*; CDR1β, *blue*; CDR2β, *limon*; CDR3β, *magenta*. *C*, percentages of TCR CDR atomic contacts with HLA-A24. Number of contacts: Total (67), CDR1α (5), CDR2α (26), CDR3α (31), CDR1β (5). *D*, interactions between TCR^NYN-I^ and the HLA-A24 α1 helix. The side chains of contacting residues are drawn in stick representation with carbon atoms in *red* (CDR3α), *blue* (CDR1β), or *gray* (HLA-A24), nitrogen atoms in *blue*, and oxygen atoms in *red*. Hydrogen bonds and salt bridge are indicated by *red* and *yellow dashes*, respectively. *E*, interactions between TCR^NYN-I^ and the HLA-A24 α2 helix. The side chains of contacting residues are drawn in *green* (CDR1α), *orange* (CDR2α), and *violet* (R65α). Hydrogen bonds are indicated by *red* dashes.

To gain insight into the molecular basis of NYN-specific TCR recognition, we chose the TCR^NYN-I^ for structural investigation. This TCR utilizes gene segments TRAV12-1 and TRAJ28 for the α chain, and TRBV6-1 and TRBJ2-7 for the β chain. We crystallized and determined the structure of TCR^NYN-I^-NYN-HLA-A24 (NYNYLYRLF) ternary complex at a resolution of 3.2 Å (Table S2). The crystal contained two complex molecules in the asymmetric unit, with a RMSD of 0.74 Å for all atoms, and we chose molecule 1 (chains A, B, E, G, and H) for our analysis (Table S4). The peptide-binding interface at the TCR: pHLA region has an unambiguous electron density in the complex structure (Fig. S5, *A*–*C*). TCR^NYN-I^ docks symmetrically over NYN-HLA-A24 in a canonical diagonal orientation, with the TCRα chain over the N terminus of the peptide and the TCRβ chain at the C-terminus (Fig. 4, *A* and *B*). TCR^NYN-I^ engaged HLA-A24 with an overall buried surface area (BSA) of 518 Å^2^, with the TCRα and TCRβ chains contributing 83% and 17% to the BSA on HLA-A24, respectively. Of a total of 67 contacts that TCR^NYN-I^ makes with HLA-A24 for MHC recognition, excluding the NYN peptide, CDR1α, CDR2α, and CDR3α contribute 7%, 39%, and 46%, respectively, compared with 7%, 0%, and 0% for CDR1β, CDR2β, and CDR3β, respectively (Fig. 4*C*). Hence, Vα dominates the interactions of TCR^NYN-I^ with MHC (62 of 67 contacts; 93%), with CDR3α contributing more to the binding interface than any other CDR. TCR^NYN-I^ makes 36 contacts with the HLA-A24 α1 helix (D85, E86, G89, K90, A93, E100) *via* CDR3α (H92α, G94α, A95α, G96α, Y98α) and CDR1β (N29β) (Fig. 4*D*). Additionally, TCR^NYN-I^ also interacts extensively with the HLA-A24 α2 helix *via* CDR1α and CDR2α, with Ser31α, Ser52α and Arg65α forming four hydrogen bonds with Glu178 and Gln179 that link the TCR to the central section of helix α2: S31α Oγ-Oε1 Q179 HLA-A24, S52α Oγ-Oε2 E178 HLA-A24, S52α N-Oε1 E178 HLA-A24, and R65 Nη2-O E178 HLA-A24 (Fig. 4*E*).

### NYN epitope recognition by TCR^NYN-I^ reveals the mechanism of immune escape

**Figure 5:**
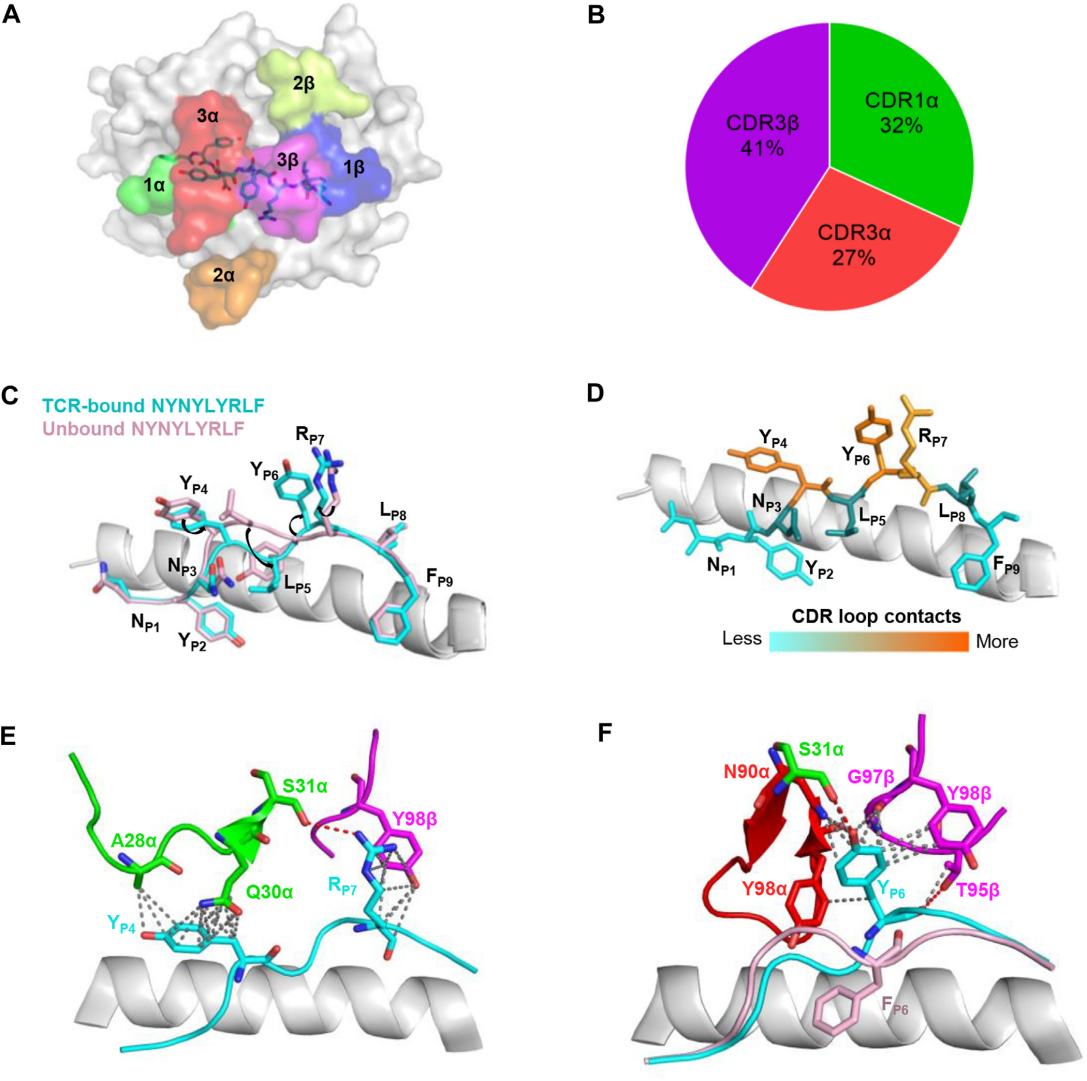
**Interactions of TCR**^**NYN-I**^**with the NYN peptide.***A*, footprint of TCR^NYN-I^ on NYN-HLA-24. The top of the HLA-A24 is depicted as a *gray* surface. *B*, percentage of TCR CDR atomic contacts with the NYN-peptide. Number of contacts: Total (66), CDR1α (21), CDR3α (18), CDR3β (27). *C*, comparison of unbound NYNYLYRLF-HLA-A24 (pink sticks, ID: 7F4W) and TCR-bound NYNYLYRLF-HLA-A24 (*cyan sticks*) peptide presentation. HLA-A24 is shown as a *gray* cartoon. *D*, heat map of TCR^NYN-I^ contacts with the NYN peptide. *E*, P4-Tyr and P7-Arg of the NYN peptide establish contacts with TCR^NYN-I^ through CDR1α (*green*) and CDR3β (*magenta*). Contacts and hydrogen bond are shown as *grey* and *red* dashes, respectively. *F*, NYNYLYRLF and NYNYL**F**RLF P6 residues are shown as *pink* and *cyan* sticks, respectively. HLA-A24 is shown as *gray* cartoon. TCR^NYN-I^ CDR1α, CDR3α, and CDR3β loops shown as *green*, *red*, and *magenta sticks*, respectively. Hydrogen bonds are shown in *red dashes*. Van der Waals contacts are shown as *grey dashes*.

As depicted by the footprint of TCR on pMHC, TCR^NYN-I^ establishes contacts with the N-terminal half of the peptide through the CDR1α and CDR3α loops, whereas the CDR3β loop mostly contacts the C-terminal half (Fig 5*A*). The contacts (66) between TCR^NYN-I^ and the NYN peptide are mediated by germline-encoded CDR1α and somatically-generated CDR3, with CDR1α, CDR3α, and CDR3β accounting for 32%, 27%, and 41%, respectively (Fig. 5*B*) (Table S5). Comparing the unbound and TCR-bound structures of the peptide revealed the residues of P4-Tyr, P5-Leu, P6-Tyr, and P7-Arg undergo a conformational shift during TCR^NYN-I^ binding (Fig. 5*C*). Analysis of the contacts between the peptide and CDR loops during binding showed that P4-Tyr, P6-Tyr, and P7-Arg accounted for 39%, 27%, and 21% of the interactions, respectively, confirming the importance of these residues for TCR recognition (Fig. 5*D*). P4-Tyr and P7-Arg mainly interact with CDR1α (A28α, Q30α, S31α) and CDR3β (Y98β) loops, anchoring the N-terminal and C-terminal of the peptide at the binding interface, respectively (Fig. 5*E*). The principal focus is on P6-Tyr, which alone contributes 18 of 66 contacts and two of five hydrogen bonds with TCR. A closer examination of the interactions facilitated by P6-Tyr showed the CDR1α, CDR3α, and CDR3β loops all make contact with this peptide residue (Fig. 5*F*). TCR^NYN-I^ CDR3β establishes major contacts with both P6-Tyr and P7-Arg of the peptide, further highlighting the crucial contribution of CDR3β to TCR^NYN-I^-NYN-HLA-A24 complex. Several CDR residues are heavily involved in interactions with the peptide: A28α, Q30α, and S31α for CDR1α, N90α, and Y98α for CDR3α, T95β, G97β, and Y98β for CDR3β (Fig. S5*D*). It is worth noting that except for T95β, the other residues are highly conserved in the five NYN-TCRs, which also explains the strong interactions observed between WT-HLA and these TCRs (Figs. S2*A*, S5*D*).

Next, we superimposed the structure of Y453F-HLA-A24 onto the TCR^NYN-I^-NYN-HLA-A24 complex using PyMol. This result suggested that the hydrophobic phenylalanine in the variants may disrupt the contacting network of the original tyrosine with TCR^NYN-I^, including the original hydrogen bonds formed between it and CDR loops (Fig. 5*F*). In addition to the Y453F mutation, our experiments indicated that L452R is another notable mutation capable of disrupting specific TCR binding. Hence, we constructed a structure model of L452R-HLA by AlphaFold2 (42) and found that the peptide fits well into the HLA-A24 groove, with the side chains of P2-Tyr and P9-Phe anchored in the pockets B and F, respectively (Fig. S5*E*). The superimposed structures of L452R-HLA-A24 and TCR^NYN-I^-NYN-HLA-A24 complex revealed that the L452R mutation might induce a significant conformational change at P4-Tyr and P5-Arg of the peptide, leading to the formation of two steric clashes with CDR3α and CDR1α, respectively (Fig. S5*F*). Our structural data elucidate how dominant mutations within the epitope, including Y453F and L452R, jeopardize the interactions between the CDR loops and the peptide, resulting in the loss of recognition by TCR^NYN-I^ T cells.

## Discussion

In T cell-mediated immune response, the TCR repertoire activated by a specific antigen epitope varies among individuals and can reach a variability of up to 10^15^. Despite the inter- and intra-individual TCR variability, several studies have described “public” TCR in COVID-19 convalescents with conserved CDR within and between individuals (18, 43, 44). For instance, HLA-A∗02:01-restricted YLQ epitope tends to elicit highly public TCRs with TRAV12-1 or TRAV12-2 genes, as exemplified by TCR YLQ7 (18). Similarly, this strong bias in V-gene usage also observed in the NYN-specific α and β chains, suggesting the importance of germline-encoded features in TCR recognition of pHLA ligands. As TRAV12-1 and TRBJ2-7 were used by 74% and 80% of NYN-specific TCRs (Fig. 1*D*), we sought to understand the molecular basis underpinning these TCR biases. TRAV12-1 encodes CDR1α Ala28 and CDR1α Ser31, whereas TRAV12-2 and TRAV12 to 3, which are nearly identical to TRAV12-1 but are not selected in the NYN-specific TCR repertoire, encode CDR1α Gly28 and CDR1α Ser31, CDR1α Ala28 and CDR1α Tyr31, respectively (Fig. S6*A*). TCR^NYN-I^ Ala28 establishes contacts with P4-Tyr of the NYN peptide, and the change of Ala28 to Gly28 of TRAV12-2 is expected to abolish the contacts with the peptide at this location (Fig. S6*B*). Moreover, the hydroxyl group of the Ser31 side chain in TCR^NYN-I^ participates in multiple polar contacts, engaging P6-Tyr and P7-Arg of the NYN peptide and the MHC residue Gln 179, and this polar network would likely not be possible with a bulkier Tyr residue of TRAV12 to 3 (Fig. S6*C*). Both Ala28 and Ser31 provide evidence for a bias towards TRAV12-1 rather than TRAV12-2 or TRAV12 to 3. Similarly, TRBJ2-7 encodes CDR3β Tyr98, whose side chain forms a hydrogen bond with P8-Leu of the peptide, and makes sufficient contact with P6-Tyr and P7-Arg of the peptide. Other TRBJ2 family members, which are not selected by the NYN-specific TCR repertoire, encode CDR3β Asn98 (TRBJ2-1, TRBJ2-4, and TRBJ2-6), Gly98 (TRBJ2-2), Asp98 (TRBJ2-3) or Glu98 (TRBJ2-5), whose smaller or missing hydroxyl group of side chains cannot replicate these key interactions (Fig. S6, *D* and *E*). Furthermore, TCR bias was also observed in the selection of TRAJ genes. More than half of the NYN-specific TRAJ genes were composed of AJ28 (19%), AJ33 (16%), AJ12 (13%), and AJ53 (10%) (Fig. 1*C*). Tyr98α, which is conserved in these four TRAJ genes, extensively makes contact with the N-terminus of the peptide (Fig. S6, *F* and *G*).

T cell escape can occur through a variety of strategies. SARS-CoV-2 variants may down-regulate the surface expression of MHC-I molecules by viral proteins such as ORF8, ORF7a, and ORF3a to evade T cell surveillance (45, 46, 47, 48). Meanwhile, amino acid changes within epitopes may affect proteasome processing (49), peptide binding to MHC (13), and TCR recognition (12, 14), leading to irreversible reductions or loss of T cell responses. SARS-CoV-2 variants are predicted to possess numerous peptides with decreased binding to typical MHC-I molecules, many of which have been experimentally validated (50). Another study found that the mutations within HLA-A∗02:01- and B∗40:01-restricted CTL epitopes resulted in weaker binding to HLA molecules, leading to diminished CD8^+^ T cell immunity (13). The P272L mutation within the YLQ killer epitope failed to activate CD8^+^ T cells in HLA-A∗02:01^+^ COVID-19 convalescents, allowing the variants to evade T cell responses (11). Unlike mutations that affect pHLA complex formation during the presentation stage, we found that L452R and Y453F mutations within the NYN epitope impact the second stage of T cell activation, the TCR recognition stage. Although mutant pHLA complexes show even higher stability than the original pHLA, the L452R and Y453F mutations led to sharp reductions in their pHLAs binding to two public TCRs, TCR^NYN-I^ and TCR^NYN-II^. Structural analysis indicated that the Y453F mutation may induce a non-functional conformation, compromising the interactions between epitope and peptide-specific TCRs. Our findings also support and provide a molecular explanation for previous discoveries that the L452R and Y453F variants contribute to virus transmission by enhancing fusion, infectiveness, and escaping from HLA-A-A24 restricted cellular immunity (28, 51).

In summary, we validated a strategy for pathogenic viruses (such as SARS-CoV-2) to escape from host CTL responses. While viral host cell entry, antigen processing and epitope presentation remain unaffected in some variants, subtle conformation changes on the peptide due to mutations diminish or even abrogate CD8^+^ T cell activation mediated by TCR recognition. This study enriches our understanding of viral mutations and their driving forces of viral evolution, which may contribute to guiding future vaccine design and therapeutic interventions targeting SARS-CoV-2 and other viral pathogens.

## Experimental procedures

### Protein preparation

Soluble pHLA proteins were prepared by in *vitro* refolding of *E. coli* inclusion bodies as our previous study (12). In brief, the MBP-β2m fusion protein was purified by affinity chromatography (0.25 M NaCl, 20 mM Tris-HCl [pH 8.0], 16–400 mM imidazole gradient) on a Hisprep IMAC column (GE Healthcare). Subsequently, the MBP tag was removed by overnight incubation with TEV protease at 4 °C. Pure β2m was obtained after further purification using secondary nickel column chromatography. HLA-A∗24:02-α was expressed as inclusion bodies in *E. coli*, which were then washed with 50 mM Tris-HCl [pH 8.0] and 5% (v/v) Triton X-100, followed by dissolution in 8 M urea, 50 mM Tris-HCl [pH 8.0]. The denatured HLA-A∗24:02-α protein was purified on a Hisprep IMAC column with an elution buffer (8 M urea, 50 mM Tris-HCl [pH 8.0], 0–0.4 M imidazole gradient). For the formation of pHLAs, a mixture of 3 mg of β2m, 2 mg of peptide, and 12 mg of HLA-A∗24:02-α was dropped into 200 ml of cold refolding buffer (5 mM reduced L-Glutathione, 5 mM oxidized L-Glutathione, 2 mM EDTA, 5% glycerol, 1% sodiumazide, 0.4 M L-Arginine, 0.1 M Tris-HCl [pH 8.0]) and incubated with magnetic stirring at 4 °C for 72 h. The refolded mixture was concentrated to a volume of 5 ml with centrifugal concentrators (membrane cutoff, 30 kDa) and further purified by a HiLoad 16/600 Superdex 200 column (GE Healthcare) with gel filtration buffer (150 mM NaCl, 20 mM HEPES [pH 7.5]). All the peptides used in this study were synthesized by the company GenScript.

Soluble TCR proteins were produced from *E. coli* inclusion bodies expressed, refolding, and purified using established procedures (14). Briefly, the TCRα (40 mg) and TCRβ (40 mg) chains were mixed and diluted into 800 ml of folding buffer (5 M urea, 0.4 M L-arginine-HCl, 3.7 mM cystamine, 6.6 mM cysteamine, 100 mM Tris-HCl [pH 8.0]), and incubated 72 h with magnetic stirring at 4 °C. The folding mixture was then dialyzed at 4 °C into a buffer containing 20 mM NaCl and 20 mM Tris-HCl [pH 8.0]. TCR heterodimers were purified using anion exchange chromatography (20 mM Tris-HCl [pH 8.0], 0–1 M NaCl gradient) and followed by a HiLoad 16/600 Superdex 200 column (GE Healthcare) for further purification.

### Surface plasmon resonance (SPR)

SPR measurements were conducted at 25 °C using a BIAcore 8K biosensor. TCRs were diluted to a concentration of 5 μg/ml with sodium acetate [pH 4.0] and immobilized onto a CM5 chip (Cytiva) using amine coupling, with the blank channel of the chip served as a negative control. All proteins were exchanged into running buffer (150 mM NaCl, 20 mM HEPES [pH 7.5] supplemented with 0.05% Tween-20). To analyze pHLAs binding, a series of different concentrations of pHLAs were sequentially flowed over chips with immobilized TCR protein. Each experiment was conducted with two biological replicates. Equilibrium dissociation constants (K_D_) were calculated by fitting kinetic data to a 1:1 binding model using Biacore evaluation software.

### Enzyme-linked immunosorbent assay (ELISA)

The pHLAs were coated on Nunc MaxiSorp plates and incubated for 16 h at 4 °C. After washing twice with PBST (PBS containing 1% Tween), the plates were blocked for non-specific binding by incubation with MPBST (PBST containing 5% skim milk) at room temperature for 1 h. Then HA-tagged TCRs were added as primary antibodies and incubated on a shaker for 3 h. After washing, anti-HA label antibody-HRP (Abmart, PA9027) was added and incubated for 3 h to detect TCR-pHLA binding. The plates were washed twice, and 100 μl TMB (Beyotime) was added to each well for 5 to 30 min. The reactions were stopped by adding 50 μl of 1 M H_2_SO_4_, and the absorbance was measured at 450 nm by Synergy H1 Tablet Reader (Biotek). Each experiment was performed in technical triplicates.

### Circular dichroism (CD)

Thermal stabilities of pHLAs loaded with WT or mutant peptides were assessed by circular dichroism (CD) spectra using a Chirascan spectrometer (Applied Photophysics). Prior to CD measurements, the sample buffer was changed to PBS, and the protein concentration was adjusted to 0.25 mg/ml. Thermal denaturation data for each protein was acquired from 25 °C to 95 °C with temperature steps of 5 °C. The data CD signals at 222 nm were extracted to calculate T_m_ values. Each experiment was performed in two biological replicates, and the data were fitted using Prism to obtain T_m_ values.

### Size-exclusion chromatography (SEC)

The formation of TCR-pHLA complexes in *vitro* was studied using gel filtration (Cytiva, Superdex-200 Increase 10/300 Gl). TCRs (TCR^NYN-I^, TCR^NYN-II^) and pHLAs (WT-HLA, L452R-HLA, Y453F-HLA, L452Q-HLA, N450K-HLA) were mixed (4 μmol of TCR mixed with 4 μmol of pHLA) in pairs and incubated for 12 h at 4 °C. Following incubation, individual pHLAs, TCRs, and their mixture were individually eluted with gel filtration buffer (150 mM NaCl, 20 mM HEPES [pH 7.5]) at a flow rate of 0.4 ml/min with AKTA pure. The formation of the TCR-pHLA complex was determined by whether the peak position of the mixture moved forward. Proteins from elution peaks were taken for SDS-PAGE detection.

### Crystallization and data collection

Purified Y453F-HLA-A24 and TCR^NYN-I^-NYN-HLA-A24 proteins were concentrated to 20 to 25 mg/ml for crystallization screening. For each protein, 0.25 μl of protein was mixed with equal-volume solutions of crystallization kits on 96-well plates and placed in an incubator at 18 °C. Crystal of Y453F-HLA-A24 was achieved in the solution containing 20% PEG 8000, 0.1 M HEPES [pH 7.5] after around 1 month, and the crystal of TCR^NYN-I^-NYN-HLA-A24 complex grew in the condition with 20% PEG3350, 8% Tacsimate [pH 7.0] after around 8 months. For data collection, crystals of Y453F-HLA-A24 and TCR^NYN-I^-NYN-HLA-A24 were immersed in a cryoprotectant consisting of 14% (v/v) ethylene glycol and 15% glycerol (v/v) with their reservoir solutions, respectively. The crystals were flashed-cooled in liquid nitrogen and sent to the Shanghai Synchrotron Radiation Facility (SSRF). X-ray diffraction data were collected at beamline BL19U1 for Y453F-HLA-A24 at the wavelength of 0.97852 Å, and BL17B1 for TCR^NYN-I^-NYN-HLA-A24 at the wavelength of 0.97854 Å.

### Structure determination and refinement

Data processing was carried out using the XDS (52) software suite. The structure was determined by molecular replacement (MR) from the CCP4 (53) program suite, using a protein databank entry (ID: 7F4W) (12) as the initial search model. Structure building was manually built *via* Coot (54) with multiple rounds of model fitting, and Phenix (55) was used for further refinement. The crystal structures were validated by the Molprobity server (56) and the RCSB ADIT validation server (57). Interactions were analyzed using the PISA server and CCP4 program. The Pymol (Schrodinger, LLC) program was used to show molecular graphics, structure superposition, and RMSD calculation between two structures.

### Statistical analysis

All of the statistical analyses and display results were performed using Prism v8 (GraphPad Software) or Excel (Microsoft) unless otherwise noted. The error bars in the plots are represented as mean ± SEM. Statistical significance (*p* values) was calculated using an unpaired Student's *t* test.

## Data availability

Crystallographic coordinates and structural factors were deposited into the RCSB PDB database (ID: 8ZV9, 8YE4). The data and information reported in this article are available upon request from the key contacts.

## Supporting information

This article contains supporting information.

## Conflict of interest

The authors declare no conflicts of interest relevant to this article.
