# Supplementary material for: Structural insights into immune escape at killer T cell epitope by SARS-CoV-2 Spike Y453F variants
Source: J Biol Chem. 2024 Jul 11;300(8):107563. doi: 10.1016/j.jbc.2024.107563 (PMC11342781; doi:10.1016/j.jbc.2024.107563)
Supplement: Supporting Information [file mmc1.pdf]

Supporting Information

Structural insights into immune escape at killer T cell epitope by SARS-CoV-2 Spike Y453F variants

Shasha Deng, Zhihao Xu, Meihua Wang, Jing Hu, Zhuan Liu, Fang Zhu, Peiyi Zheng, Arnaud John Kombe Kombe, Hongliang Zhang, Songquan Wu, Tengchuan Jin.

Supporting Figures 1-6

Supporting Tables 1-5

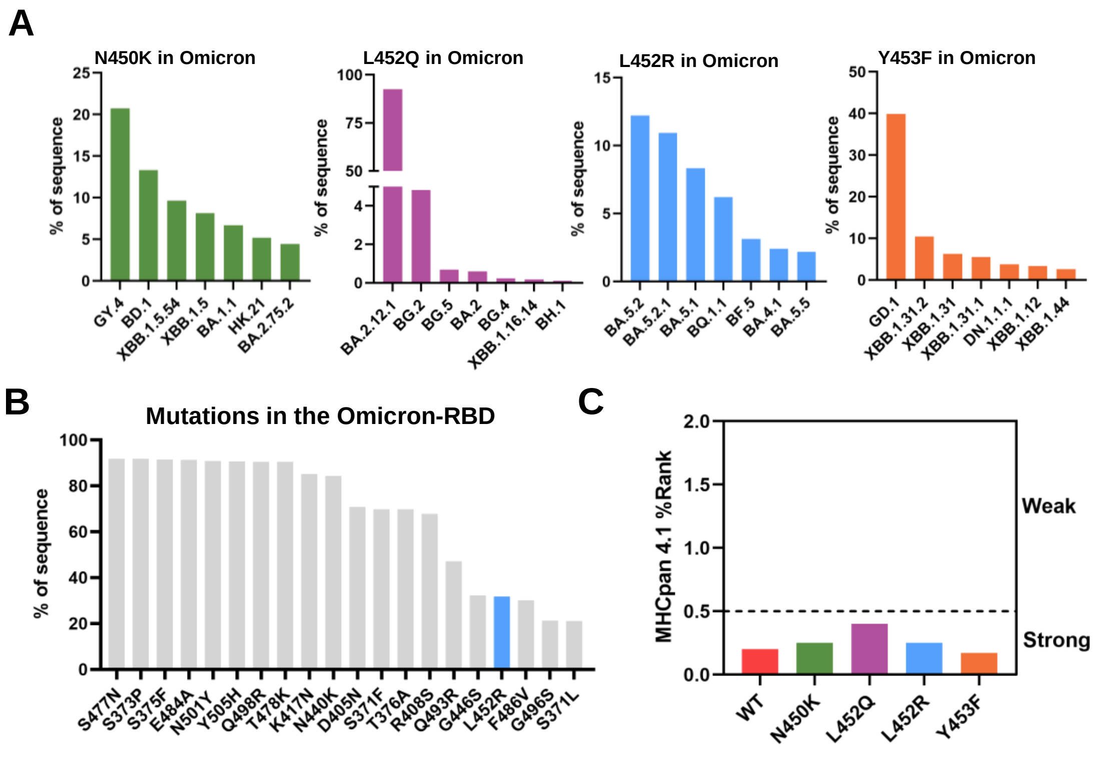

Figure S1. Mutation analysis of the NYN epitope. A, as of February 1st 2024, the percentages of several Omicron variants with NYN epitope mutations (N450K, L452Q, L452R, and Y453F). Data used in SARS-CoV-2 mutations analysis is available from the public databases of GISAID (https://www.gisaid.org) and covSPECTRUM (https://cov-spectrum.org). **B,** the top 20 mutations most frequently observed in the Omicron-RBD as of February 1st 2024. **C,** NetMHCpan4.1 binding predictions (https://services.healthtech.dtu.dk/services/NetMHCpan-4.1) for WT or mutant NYN-peptides to the HLA-A*24:02 genotype.

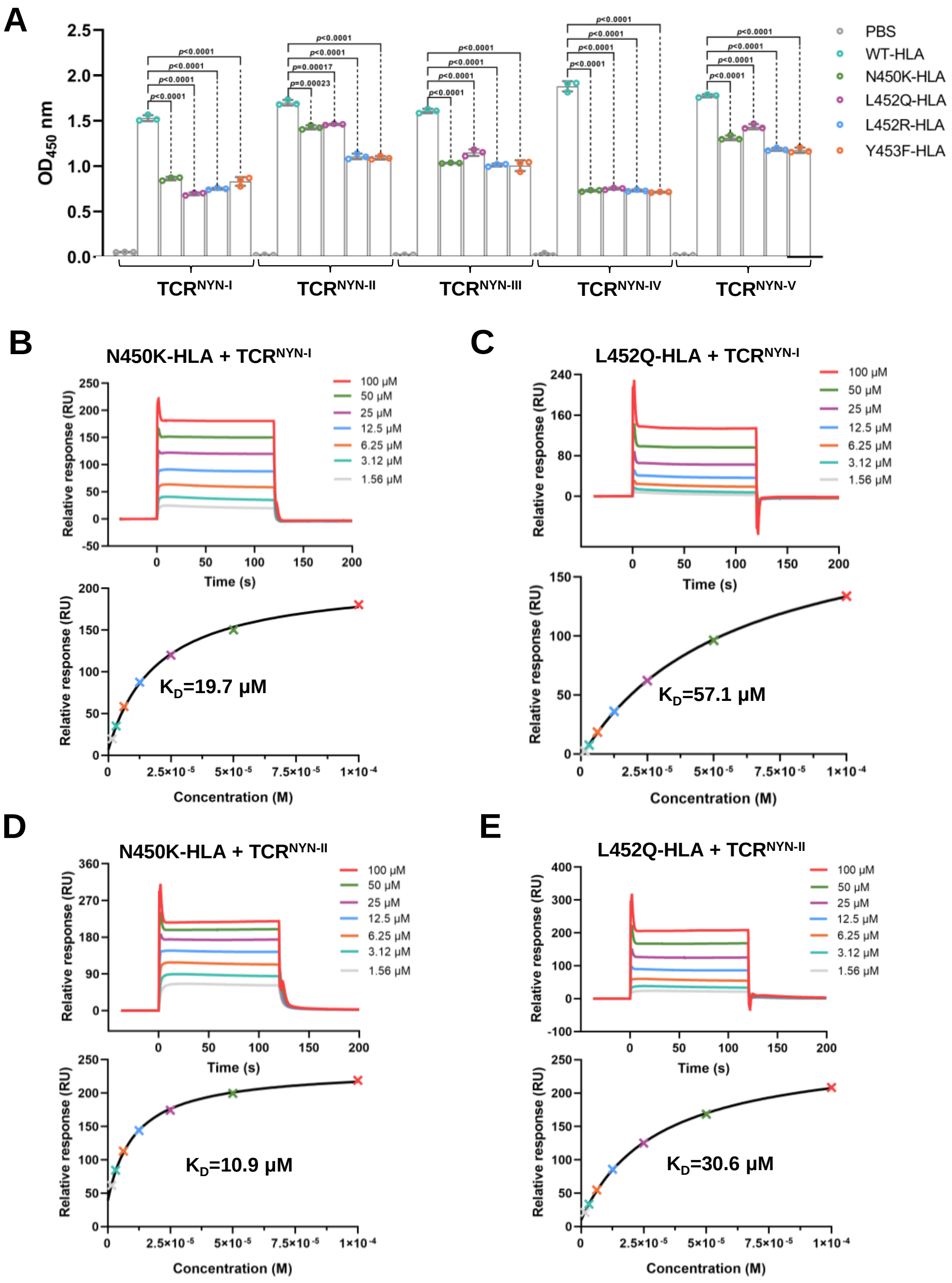

Figure S2. Detection of the decreased TCR: pMHC interaction due to mutations. A, ELISA affinity diagrams of five NYN-TCRs with WT or mutant pHLAs. Each experiment was performed in technical triplicates. The data were shown as the mean ± SEM. Statistical significance was calculated using a two-tailed unpaired Student's t test. **B-C,** (upper) N450K-HLA or L452Q-HLA at concentrations of 1.56, 3.12, 6.25, 12.5, 25, 50 and 100 μM were injected over immobilized TCR^{NYN-I}. (lower) Fitting curves for equilibrium binding that resulted in K_Ds of 19.7 μM and 57.1 μM, respectively. **D-E,** (upper) N450K-HLA or L452Q-HLA at concentrations of 1.56, 3.12, 6.25, 12.5, 25.0, 50.0 and 100 μM were injected over immobilized TCR^{NYN-II}. (lower) Fitting curves for equilibrium binding that resulted in K_Ds of 10.9 μM and 30.6 μM, respectively. Each SPR experiment was conducted with two independent biological replicates, and the repeated results were shown in **Figure S3**.

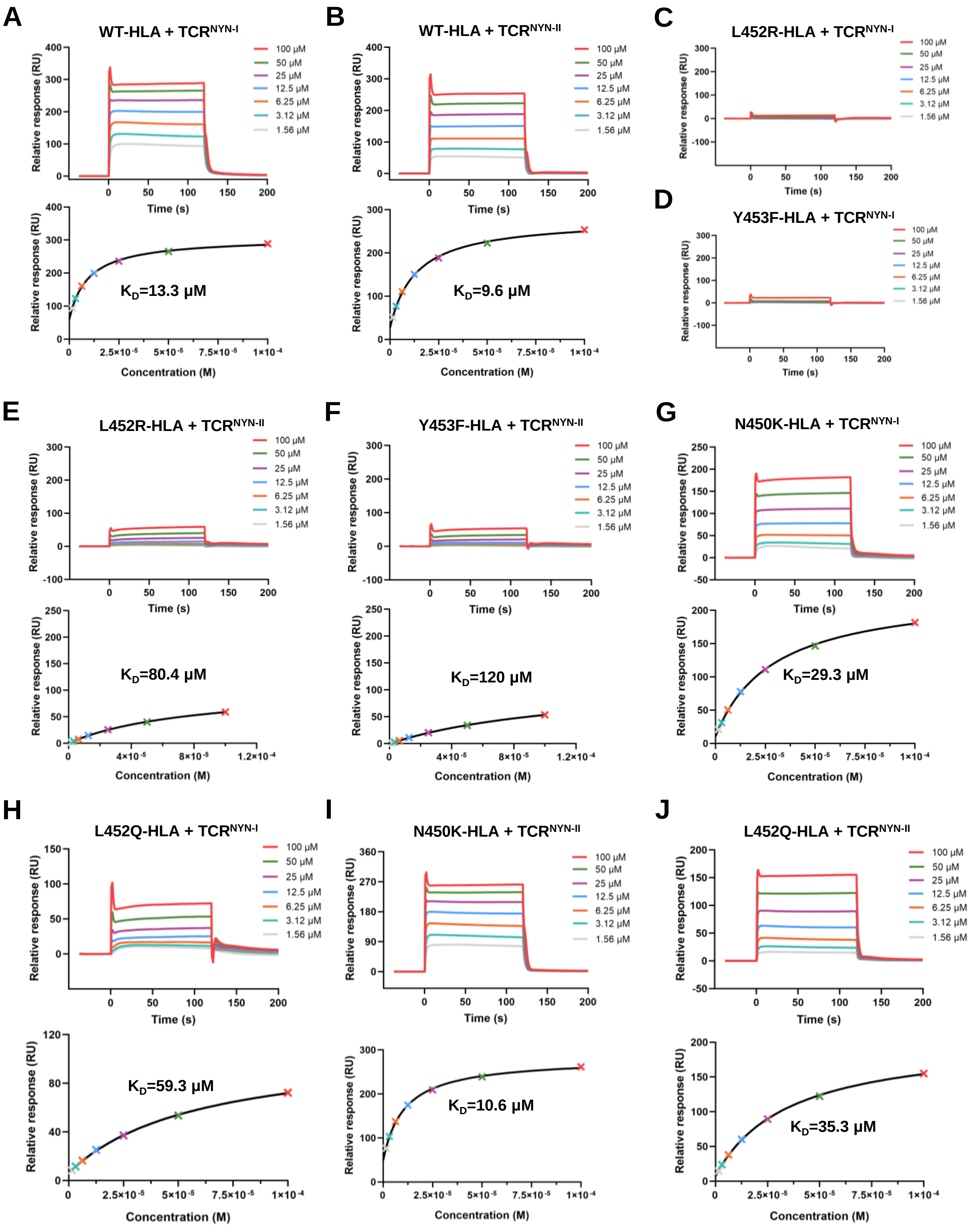

Figure S3. The repeated results of SPR experiments. A-J, WT or mutant pHLA proteins at concentrations of 1.56, 3.12, 6.25, 12.5, 25, 50 and 100 μM were injected over immobilized TCR^{NYN-I} or TCR^{NYN-II}. Each SPR experiment was conducted with two biological replicates, with the other replicate shown in Figure 2 (A-F) and Figure S2 (B-D).

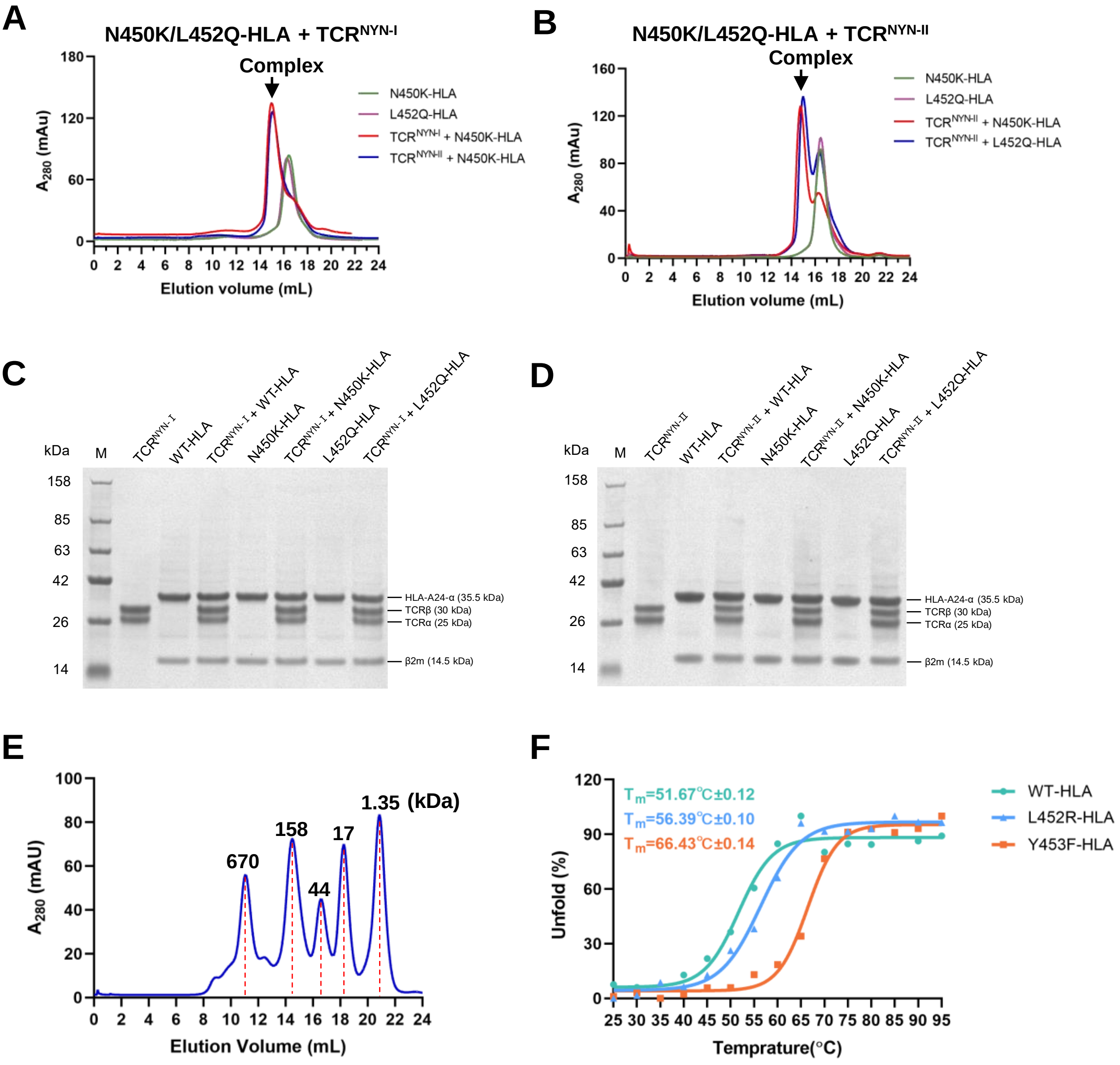

Figure S4. Additional assays of size exclusion chromatography (SEC) and circular dichroism (CD). A, SEC assays of N450K- or L452Q-HLA with TCR^{NYN-I}. **B,** SEC assays of N450K- or L452Q-HLA with TCR^{NYN-II}. **C-D,** SDS-PAGE detections of SEC assays. TCR-pHLA complex consists of 4 bands, including heavy (~35.5 kDa) and light (β2m, ~14.5 kDa) chains of pHLA, and the α (~25 kDa) and β (~30 kDa) chains of TCR. **E,** curve of standard for SEC assays. **F**, the repeated results of CD measurements. Each CD experiment was conducted with two biological replicates, with the other replicate shown in Figure 3F.

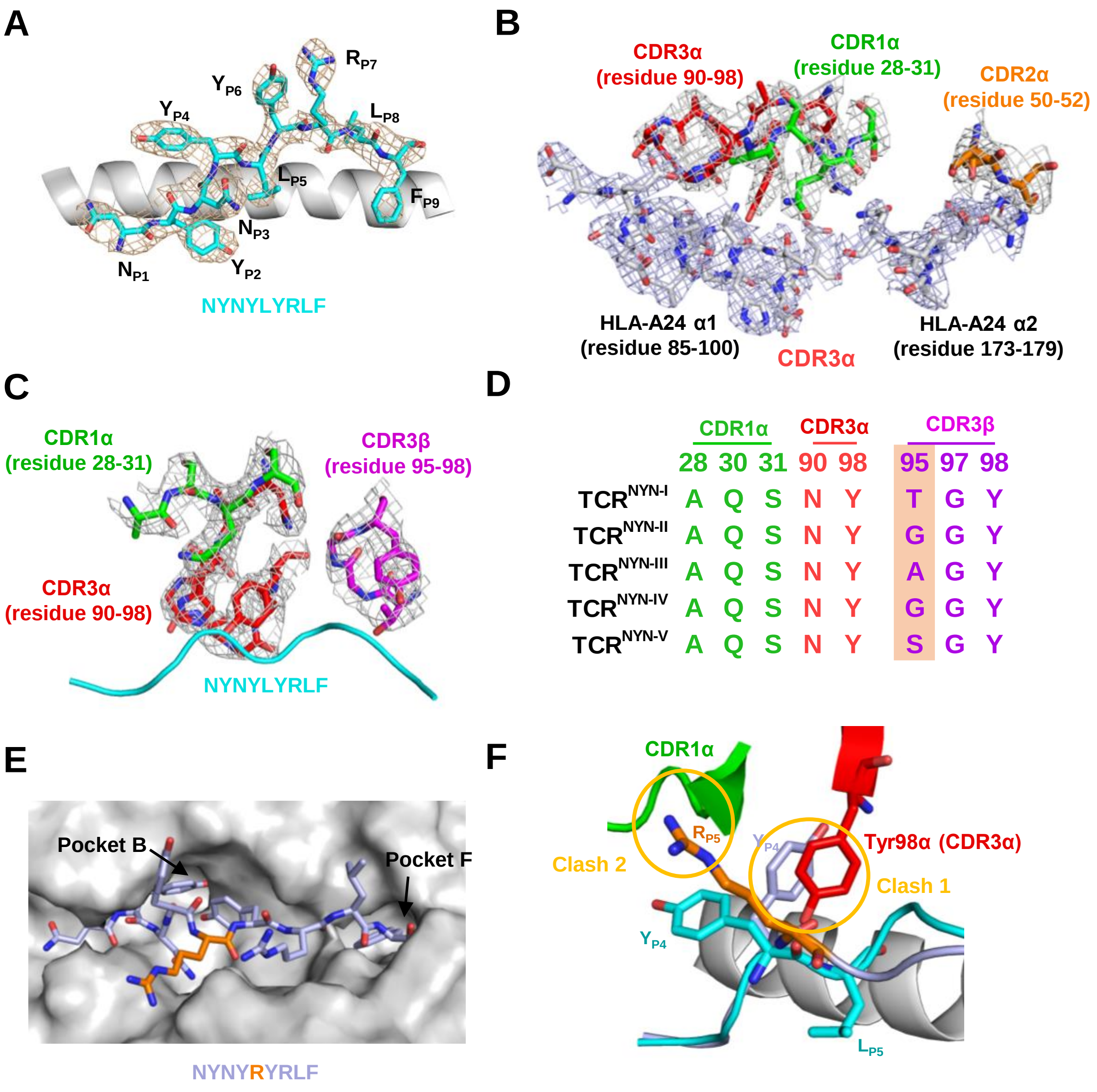

Figure S5. Supplementary figures for the crystal structure of TCR$^{\text{NYN-I}}$-NYN-HLA-A24 complex. A, electron density (2Fo-Fc) of the NYN-peptide (NYNYLYRLF) in the crystal structure of TCR$^{\text{NYN-I}}$-NYN-HLA-A24 complex. Carbon atoms of the peptides are cyan; nitrogen atoms are blue; oxygen atoms are red. HLA-A24 helix is grey. **B,** electron density (2Fo-Fc) at the interface between TCR$^{\text{NYN-I}}$ and HLA-A24. **C,** electron density (2Fo-Fc) at the interface between TCR$^{\text{NYN-I}}$ and the peptide. **D,** sequence alignment of TCR residues at key positions for the peptide: TCR interactions. **E,** peptide-binding structure of L452R-HLA predicted by AlphaFold2. The L452R peptide bind HLA-A24 in a conventional direction with side chains of P2-Tyr and P9-Phe accommodated in pockets B and F, respectively. HLA-A24 is shown in grey surface. **F,** structural superimposition of L452R-HLA-A24 and TCR$^{\text{NYN-I}}$-NYN-HLA-A24 complex. The original and L452R peptide are cyan and light blue, respectively. The mutation site of P6-Arg is highlighted in orange. P4-Tyr and P5-Arg of the peptide formed two steric clashes with CDR3α and CDR1α, respectively.

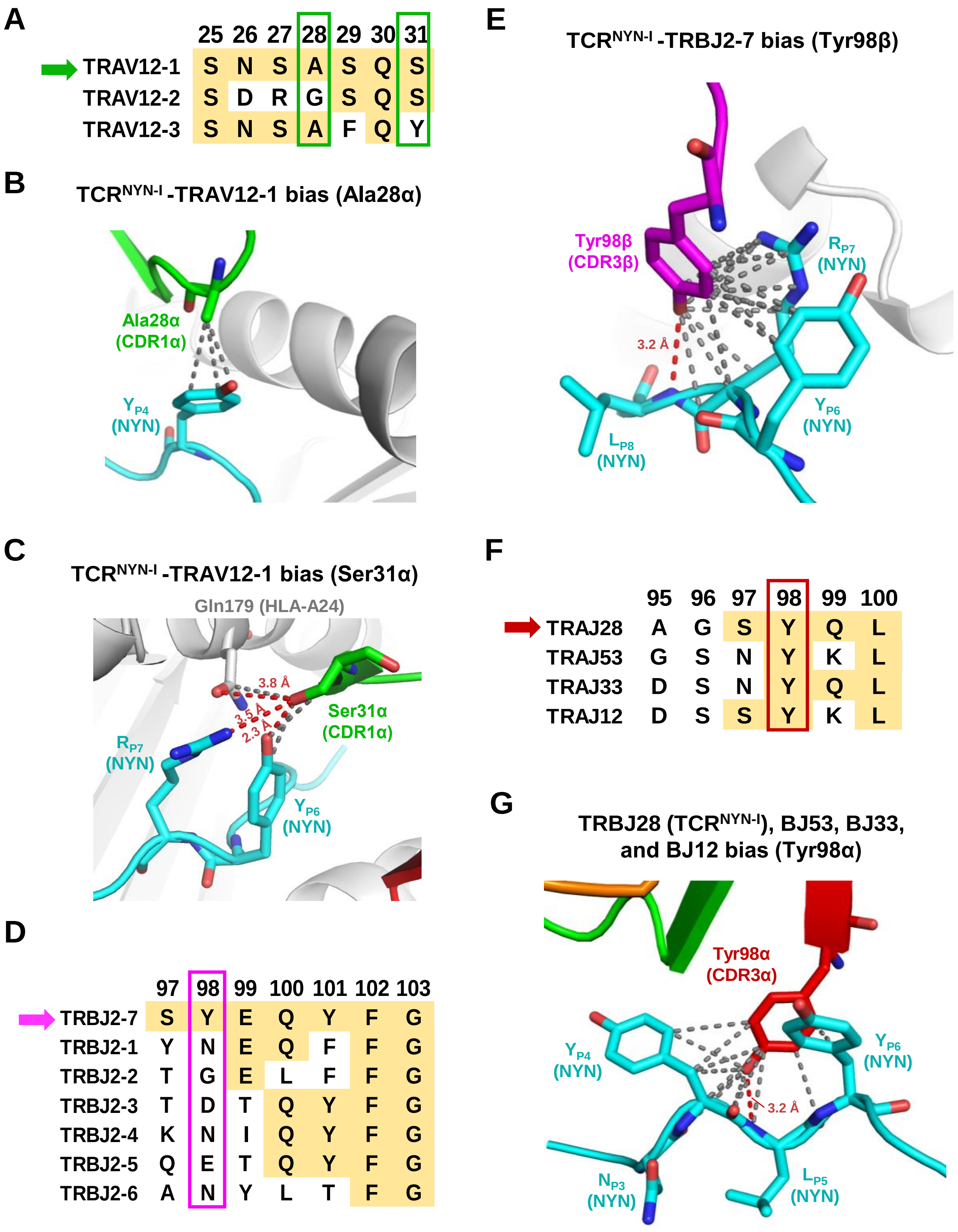

Figure S6. Structural analysis for TCR biases of NYN-TCRs. A, sequence alignment of CDR1α of TRAV12 family members. Green arrow indicates TRAV12-1 of TCR$^{NYN\text{-}I}$. **B,** Ala28α of TRAV12-1 forms three contacts with P4-Tyr of the NYN peptide. Contacts are shown as grey dashes. **C,** Ser31α of TRAV12-1 forms six contacts including three hydrogen bonds with Gln179 of HLA-A24, P6-Tyr and P7-Arg of the NYN peptide. Hydrogen bonds are shown in red dashes. Van der Waals contacts are shown as grey dashes. **D,** sequence alignment of TRBJ2 family members. Magenta arrow indicates TRBJ2-7 of TCR$^{NYN\text{-}I}$. **E,** Tyr98β of TRBJ2-7 forms eighteen contacts with P6-Tyr, P7-Arg, and P8-Leu of the NYN peptide. **F,** sequence alignment of TRAJ28, TRAJ53, TRAJ33, and TRAJ12. Red arrow indicates TRAJ28 of TCR$^{NYN\text{-}I}$. **G,** Tyr98α of TRAJ28, TRAJ53, TRAJ33, and TRAJ12 establishes fourteen contacts with P3-Asn, P4-Tyr, P5-Leu, and P6-Tyr of the NYN peptide.

Table S1. SPR summary of pHLAs binding to $TCR^{NYN\text{-}I}$ or $TCR^{NYN\text{-}II}$.

NYN-HLA	Peptide sequence	Variants	$TCR^{NYN\text{-}I}$ K_D1 (μM)	$TCR^{NYN\text{-}I}$ K_D2 (μM)	$TCR^{NYN\text{-}II}$ K_D1 (μM)	$TCR^{NYN\text{-}II}$ K_D2 (μM)
WT-HLA	NYNYLYRLF	NA	**13.5**	**13.3**	**8.6**	**9.6**
N450K-HLA	NY**K**YLYRLF	B.1.214.2, Omicron	19.7	29.3	10.9	10.6
L452Q-HLA	NYNY**Q**YRLF	Lambda, Omicron	57.1	59.3	30.6	35.3
L452R-HLA	NYNY**R**YRLF	Delta, Omicron	*	*	**74.6**	**80.4**
Y453F-HLA	NYNYL**F**RLF	Mink, Omicron	*	*	**116**	**120**

NA: not available.
*: no measurable binding was observed.

Table S2. X-ray data collection and refinement statistics.

Name	Y453F-HLA-A24	TCR^{NYN-I}-NYN-HLA-A24
**Data Collection**		
Space group	$P2_12_12_1$	$P2_12_12_1$
Unit cell (a, b, c) (Å)	47.06, 167.49, 168.58	69.00, 148.10, 196.21
(α,β,γ) (°)	90, 90, 90	90, 90, 90
Resolution (Å) (Last shell)	45.33-2.6<br>(2.66- 2.6)	39.98-3.2<br>(3.29-3.2)
No of reflections (total/unique)	536136/42070	447941/34048
Redundancy (last shell)	14.4 (11.6)	7.0 (6.8)
Completeness (%)	99.9 (99.6)	99.9 (99.9)
I/σ (I) (last shell)	18.43 (2.07)	11.06 (1.66)
Rmeas (last shell) (%)¶	11.1 (123)	41.8 (247.1)
CC1/2 (%)	99.9 (84.5)	99.1 (74.2)
**Refinement**		
Resolution (Å)	45.33-2.6	39.98-3.2
Number of protein atoms	9691	12294
Rmsd bond lengths (Å)	0.005	0.003
Rmsd bond angles (°)	0.98	0.62
R_{work} (%)†	25.81	26.79
Rfree (%)‡	28.45	31.21
Ramachandran plot favored/disallowed (%)**	97/0.09	94/0
PDB code	8ZV9	8YE4

Values in the parentheses correspond to the last resolution shell.

¶ R_{meas} = $S_h(n/n-1)^{1/2}S_i$ $|I_i(h)$ -<$I(h)$> | / S_hS_i $I_i(h)$, where $I_i(h)$ and <$I(h)$> are the ith and mean measurement of the intensity of reflection h.

† R_{work} = $S_h||F_{obs}$ (h)|-|F_{calc} (h)|| / $S_h|F_{obs}$ (h)|, where F_{obs} (h) and F_{calc} (h) are the observed and calculated structure factors, respectively. No I/s cutoff was applied.

‡R_{free} is the R-value obtained for a test set of reflections consisting of a randomly selected 5% subset of the data set excluded from refinement.

**Values from Molprobity server (http://molprobity.biochem.duke.edu/).

Table S3. Interactions between WT/Y453F peptide and HLA-A24.

NYNYLYRLF	HLA-A24	
	Hydrogen bonds (12)	Salt Bridge (1)
N_{P1}	L_{P1} (N) - Y31 (Oη)<br>L_{P1} (O) - Y183 (Oη)<br>L_{P1} (N) - Y195 (Oη)<br>L_{P1} (Oδ1) - Y195 (Oη)<br>**L_{P1} (Oδ1) - R194 (Nη2)**	
Y_{P2}	Y_{P2} (N) - Y31 (Oη)<br>Y_{P2} (N) - E87 (Oε1)<br>**Y_{P2} (Oη) - H94 (Nδ1)**	
Y_{P6}	**Y_{P6} (Oη) - K90 (O)**	
R_{P7}	R_{P7} (O) - Q101 (Nδ2)	
L_{P8}	L_{P8} (O) - W171 (Nε1)	
F_{P9}	F_{P9} (N) - Q101 (Oδ1)	F_{P9} (O) - K170 (Nζ)
NYNYLFRLF	HLA-A24	
	Hydrogen bonds (17)	Salt Bridge (1)
N_{P1}	L_{P1} (N) - Y31 (Oη)<br>L_{P1} (O) - Y183 (Oη)<br>L_{P1} (N) - Y195 (Oη)<br>L_{P1} (Nδ2) - Y195 (Oη)	
Y_{P2}	Y_{P2} (N) - Y31 (Oη)<br>Y_{P2} (N) - E87 (Oε1)<br>**Y_{P2} (N) - E87 (Oε2)**	
N_{P3}	**N_{P3} (Nδ2) - Q180 (Oε1)**<br>**N_{P3} (Oδ1) - Q180 (Nε2)**	
Y_{P4}	**Y_{P4} (Oη) - T187 (Oγ1)**	
Y_{P6}	**Y_{P6} (O) - T97 (Oγ1)**	
R_{P7}	R_{P7} (O) - Q101 (Nδ2)<br>**R_{P7} (Nε) - Q179 (Oε1)**<br>**R_{P7} (Nη2) - Q179 (Oε1)**	
L_{P8}	L_{P8} (O) - W171 (Nε1)	
F_{P9}	F_{P9} (N) - Q101 (Oδ1)<br>**F_{P9} (O) - T167 (Oγ1)**	F_{P9} (O) - K170 (Nζ)

Hydrogen bonds and salt bridges were calculated using the Protein Interfaces, Surface, and Assemblies (PISA) server with a cutoff distance of 4.0 Å. The unique bonds for WT and Y453F peptides are highlighted in bold black.

Table S4. Interactions between TCR$^{NYN\text{-}I}$ and HLA-A24.

HLA-A24	TCR$^{NYN\text{-}I}$		
	Hydrogen bonds	Van der Waals contacts (number)	Salt Bridges
α1 helix			
D85		G94 (1)	
E86	E86 (Oε2) - H92 (Nδ1)	H92 (8)<br>G94 (1)	E86 (Oε1) - H92 (Nδ1)
G89		H92 (4)<br>A95 (4)<br>G96 (3)	
K90		H92 (6)	
A93		G96 (3)<br>Y98 (1)	
E100	E100 (Oε1) - N29 (Nδ2)	N29 (5)	
α2 helix			
A173		Y50 (2)	
A174		Y50 (4)	
H175		Y50 (13)	
E178	E178 (Oε1) - S52 (N)<br>E178 (Oε2) - S52 (Oγ)<br>E178 (O) - R65 (Nη2)	Y50 (4)<br>S51 (1)<br>S52 (2)<br>R65 (1)	
Q179	Q179 (Oε1) - S31 (Oγ)	Q30 (3)<br>S31 (2)	

Hydrogen bonds, salt bridges and Van der Waals contacts were calculated using the Protein Interfaces, Surface, and Assemblies (PISA) server and CCP4 program with a cutoff distance of 4.0 Å.

Table S5. Interactions between TCR^{NYN-I} and NYN peptide.

NYNYLYRLF	TCR^{NYN-I}	
	Hydrogen bonds	Van der Waals contacts (number)
N_{P3}		Y98α (1)
Y_{P4}		Y98α (9)<br>Q30α (14)<br>A28α (3)
L_{P5}	L_{P5} (N) - Y98α (Oη)	Y98α (3)
Y_{P6}	Y_{P6} (Oη) - S31α (Oγ)<br>Y_{P6} (O) - T95β (Oγ1)	Y98α (1)<br>N90α (4)<br>S31α (3)<br>T95β (2)<br>G97β (4)<br>Y98β (4)
R_{P7}	R_{P7} (Nη1) - S31α (Oγ)	S31α (1)<br>Y98β (13)
L_{P8}	L_{P8} (N) - Y98β (Oη)	Y98β (4)

Hydrogen bonds and Van der Waals contacts were calculated using the Protein Interfaces, Surface, and Assemblies (PISA) server and CCP4 program with a cutoff distance of 4.0 Å.
